# Effect of Pod e-Cigarettes vs Cigarettes on Carcinogen Exposure Among African American and Latinx Smokers: A Randomized Clinical Trial

**DOI:** 10.1001/jamanetworkopen.2020.26324

**Published:** 2020-11-18

**Authors:** Kim Pulvers, Nicole L. Nollen, Myra Rice, Christopher H. Schmid, Kexin Qu, Neal L. Benowitz, Jasjit S. Ahluwalia

**Affiliations:** 1 Department of Psychology, California State University, San Marcos; 2 Department of Population Health, University of Kansas School of Medicine, Kansas City; 3 Department of Biostatistics, School of Public Health, Brown University, Providence, Rhode Island; 4 Program in Clinical Pharmacology, Division of Cardiology, Department of Medicine, University of California School of Medicine, San Francisco; 5 Center for Alcohol and Addiction Studies, Department of Behavioral and Social Sciences, School of Public Health, Department of Medicine, Alpert Medical School, Brown University, Providence, Rhode Island

## Abstract

**Importance:**

Fourth-generation nicotine salt pod system (NSPS) electronic cigarettes (e-cigarettes) are the leading class of e-cigarettes. They contain high nicotine concentrations, which may facilitate switching among smokers, but could also lead to increased exposure to nicotine and biomarkers of potential harm. African American and Latinx smokers experience significant tobacco-related health disparities. The potential of NSPS e-cigarettes to reduce smoking-related harm among these groups is unknown.

**Objective:**

To compare the harm reduction potential of NSPS e-cigarette vs combustible cigarettes.

**Design, Setting, and Participants:**

This unblinded randomized clinical trial compared 6 weeks of e-cigarette use vs cigarettes as usual from to 2018 to 2019 among smokers in the San Diego, California, and Kansas City, Missouri, areas. Participants included African American and Latinx adult combustible cigarette smokers who smoked at least 5 cigarettes/d on at least 25 of the past 30 days for at least 6 months and were interested in switching to e-cigarettes. Data were analyzed from September 18, 2019, to September 4, 2020.

**Interventions:**

6 weeks of e-cigarette use in a choice of pod flavors (5% nicotine) along with brief education, training, and action planning to completely switch to e-cigarettes from combustible cigarettes. The control group smoked combustible cigarettes as usual.

**Main Outcomes and Measures:**

The primary outcome was reduction in urinary 4-(methylnitrosamino)-1-(3-pyridyl)-1-butanol (NNAL) concentration at week 6. Secondary outcomes were change in urinary cotinine, expired carbon monoxide (CO), respiratory symptoms, lung function, blood pressure, past 7-day consumption of combustible cigarettes, and switching rates (e-cigarette group only) at weeks 2 and 6.

**Results:**

This study included 186 participants, including 92 African American participants and 94 Latinx participants. The mean (SD) age was 43.3 (12.5) years, and 75 (40.3%) were women. Participants smoked a mean (SD) of 12.1 (7.2) cigarettes/d on 6.8 (0.6) d/wk at baseline. A total of 125 participants were randomized to the e-cigarette group and 61 were randomized to the control group. At baseline, median (interquartile range) NNAL was 124 (45-197) pg/mL in the e-cigarette group and 88 (58-197) pg/mL in the control group. At week 6, the e-cigarette group had significantly greater reductions in NNAL (relative risk [RR], 0.36 [95% CI, 0.23-0.54]; *P* < .001), CO (RR, 0.53 [95% CI, 0.42-0.68]; *P* < .001), respiratory symptoms (RR, 0.63 [95% CI, 0.47-0.85]; *P* = .002), and number of cigarettes smoked in the past 7 days among those still smoking (RR, 0.30 [95% CI, 0.20-0.43]; *P* < .001) than the control group and maintained their cotinine levels (RR, 0.80 [95% CI, 0.58-1.10]; *P* = .17). Lung function and diastolic and systolic blood pressure remained unchanged and did not differ between groups. For participants randomized to receive e-cigarettes, 32 participants (28.1%) were exclusively using e-cigarettes at week 6, while 66 participants (57.9%) were dual using and 16 participants (14%) resumed exclusively using cigarettes.

**Conclusions and Relevance:**

These findings suggest that e-cigarettes may be an inclusive harm reduction strategy for African American and Latinx smokers.

**Trial Registration:**

ClinicalTrials.gov Identifier: NCT03511001

## Key Points

**Question:**

What is the effect of using nicotine salt pod system (NSPS) electronic cigarettes (e-cigarettes) for replacing cigarettes (ie, switching) on biomarkers of tobacco exposure and potential harm among cigarette smokers?

**Findings:**

In this randomized clinical trial including 186 African American and Latinx adult smokers, those randomized to the e-cigarette condition maintained their cotinine levels and significantly reduced urinary 4-(methylnitrosamino)-1-(3-pyridyl)-1-butanol (NNAL) and carbon monoxide levels and respiratory symptoms compared with controls smoking as usual at week 6. Lung function and blood pressure remained unchanged.

**Meaning:**

This randomized clinical trial found that switching to NSPS e-cigarettes among adult smokers did not increase nicotine exposure and led to short-term reduction in the major pulmonary carcinogen, NNAL, compared with continued smoking.

## Teaser

This randomized clinical trial examines biomarkers of carcinogen exposure in African American and Latinx smokers who switch to electronic cigarettes vs smoking as usual.

## Introduction

The risk-benefit tradeoff of electronic cigarettes (e-cigarettes) has divided the medical and public health communities.^1^ Fourth-generation nicotine salt pod system (NSPS) e-cigarettes are the current market leaders.^2,3^ These devices contain nicotine as the benzoate or other salt in relatively high concentrations and use a low wattage battery. For example, JUUL liquid (JUUL Labs) contains 5% nicotine by weight, equivalent to approximately 59 mg/mL nicotine vs 0 to 36 mg/mL in non-NSPS e-cigarette liquids. These features provide efficient nicotine delivery with minimal inhalation irritation in a compact device that resembles a flash drive and charges in a magnetic USB port.^4,5^

High nicotine delivery and other reinforcing features of fourth-generation NSPS e-cigarettes have led to significant uptake among adolescents,^6,7^ but these features may also support switching and potential harm reduction among adult combustible cigarette smokers. Little is known about the benefits and adverse effects of NSPS e-cigarette use in adult combustible cigarette smokers. The National Academies of Science, Engineering, and Medicine 2018 report^8^ concluded that, when used exclusively, e-cigarette pose significantly less exposure to toxicants and less short-term health risks than combustible cigarettes. Studies included in the 2018 report^8^ used first-, second-, and third-generation e-cigarettes.

To our knowledge, this study is the first randomized clinical trial to examine biomarkers of exposure and potential harm of switching to a leading fourth-generation e-cigarette (e-cigarette group) vs continuing to smoke cigarettes as usual (control group). Members of the 2 largest minority groups in the US who have been underrepresented in previous e-cigarette studies, African American and Latino/Latina (hereafter, *Latinx*) smokers,^8^ were the focus, given their high rates of tobacco-related morbidity and mortality at lower levels of smoking^9,10^ and their being less likely than White smokers to switch to exclusive e-cigarette use.^11,12^ Racial and ethnic disparities in exclusive switching to e-cigarette could exacerbate the greater burden of tobacco-related death and disease among disadvantaged populations.^13^

The primary hypothesis was that carcinogen exposure, measured via 4-(methylnitrosamino)-1-(3-pyridyl)-1-butanol (NNAL; a pulmonary tobacco-specific carcinogen) from baseline to week 6 would be significantly lower in the e-cigarette group compared with a smoking-as-usual control group. Additional short-term outcomes included change in cigarette consumption, urinary cotinine, expired carbon monoxide (CO), respiratory symptoms, lung spirometry, and blood pressure (BP). We also examined within-group differences in these factors and by e-cigarette use trajectory (ie, exclusive e-cigarette, dual e-cigarette and cigarette, and exclusive cigarette) in the e-cigarette group. These findings could provide critical information to guide regulatory and policy decisions, along with needed medical advice.

## Methods

This unblinded randomized clinical trial compared toxicant exposure in smokers randomized to 6 weeks of e-cigarette use vs continuing to smoke cigarettes as usual. Recruitment occurred from May 10, 2018, through March 29, 2019, with follow-up completed by May 17, 2019. The study was approved by the institutional review board at California State University, San Marcos, and University of Kansas School of Medicine. All participants provided written informed consent. The study protocol, including recruitment methods, are provided in the Trial Protocol in Supplement 1. This study is reported following the Consolidated Standards of Reporting Trials (CONSORT) reporting guideline.

### Participants and Setting

Participants were recruited from the San Diego, California, and Kansas City, Missouri and Kansas, metropolitan areas. Participants were eligible if they were aged 21 years or older, smoked at least 5 cigarettes per day on at least 25 of the past 30 days, smoked cigarettes for at least 6 months, had expired CO of greater than 5 ppm at baseline, had systolic BP of less than 160 mm Hg and diastolic BP of less than 105 mm Hg at baseline, self-identified as Hispanic or Latinx in San Diego or Black or African American in Kansas City, were fluent in English or Spanish, and were willing to switch from smoking cigarettes to e-cigarettes for 6 weeks.

Participants were excluded if they primarily used other tobacco products or equally used cigarettes and other tobacco products, used e-cigarette on 4 or more of the past 30 days, were currently enrolled in a smoking cessation program or other clinical trial, used smoking cessation pharmacotherapy in the past 30 days, had been hospitalized for mental illness in the past 30 days, had a heart-related illness in the past 30 days, resided with another person enrolled in the study, were planning to move away from San Diego or Kansas City during the study period, had unstable mental status or health status, or were pregnant, breastfeeding, or planning to become pregnant in the next 6 months.

### Randomization

Participants were randomly assigned in a 2:1 ratio to facilitate more experience with this novel intervention given no significant changes expected in the control group,^14,15^ stratified by study site (African Americans in Kansas City and Latinx in San Diego), to e-cigarette or cigarettes as usual. The randomization sequence was generated with an Excel (Microsoft) random number formula applied to each site. Allocation was placed into sealed individual envelopes labeled with participant identification numbers for each site, retrieved from a locked cabinet monitored by the project manager, and opened individually following consent of each participant.

### Intervention

Those randomized to the e-cigarette group received a JUUL e-cigarette and pods in a choice of flavor (5% nicotine), along with brief education, training, and action planning for making a complete switch to e-cigarettes. Choice of pod flavors is shown in eTable 1 in Supplement 2. Allocation of pods was 1 pod per pack of cigarettes. A 2-week supply of pods was provided at baseline and an additional 4 weeks of pods were provided at the week 2 visit. At each follow-up appointment (week 1, telephone call; week 2, in-person visit; and week 4, telephone call), barriers and benefits of switching to e-cigarette were discussed and action planning for exclusive switching was revisited. Participants in the control group received assessment only and continued smoking as usual.

### Outcomes and Measures

Participants completed assessments at baseline, week 2, and week 6 and were compensated on a schedule of $20 at baseline, $40 at week 2, and $60 at week 6. Data were entered into a REDCap database (Vanderbilt University) and audited by the project manager. Descriptive variables included sex, age, race/ethnicity, education level, income, and marital status. Individuals who identified as Latinx were classified as such regardless of race. Participants were asked whether they usually smoked menthol or nonmenthol cigarettes, how long they had smoked cigarettes, and usual time to first daily cigarette, as smoking within 30 minutes of waking is considered an indicator of higher nicotine dependence.^16,17^

The primary outcome was reduction in toxicant exposure, as measured by NNAL excretion. Urine NNAL concentration reflects exposure to the tobacco specific nitrosamine and lung carcinogen, 4-(methylnitrosamino)-1-(3-pyridyl)-1-butanone. Urine samples were tested for concentrations of NNAL measured by ultraperformance liquid chromatography–tandem mass spectrometer and normalized for creatinine.^18,19^ Limit of quantification was 30 pg/mL. Absolute values for below limit of quantification results, which may vary 20% from actual concentration, were used (16 values at baseline; 47 values at week 6).

Secondary outcomes included change in past 7-day combustible cigarette use measured by 7-day timeline follow-back interview^20,21,22^; cotinine, the main proximate metabolite of nicotine (measured from urine samples by ultraperformance liquid chromatography–tandem mass spectrometer and normalized for creatinine)^18,19^; CO (measured via Micro+Smokerlyzer [coVita]), an exposure measure of combusted tobacco; lung function as the mean midexpiratory phase of forced expiratory (FEF_25%-75%_) (measured via Discovery-2 spirometer [SpiroVision]), the pulmonary function test of small airway disease that is most sensitive to effects of cigarette smoking^23^; respiratory symptoms as measured with the American Thoracic Society Questionnaire (scores range from 0-32, with higher scores indicating greater respiratory symptoms)^24,25^; and BP (measured via BP742N 5 Series digital BP cuff [Omron]). Researchers were trained to competency on administering all measures.

e-Cigarette use trajectories were quantified among the e-cigarette group separately at weeks 2 and 6. Exclusive e-cigarette users were defined as individuals who reported any use of e-cigarettes and no use of cigarettes in the past 7 days and who had CO level less than 6 ppm. Dual users were defined as individuals who reported any use of e-cigarettes and any use of cigarettes in the past 7 days. Additionally, individuals who reported no use of cigarettes in the past 7 days but who had a CO level of 6 ppm or greater were conservatively classified as dual users. Exclusive cigarette smokers were defined as individuals who reported no use of e-cigarettes and any use of cigarettes in the past 7 days.

A 6-month follow-up survey was conducted by telephone with individuals in the e-cigarette group. Past 30 days use of cigarettes and e-cigarettes was assessed. e-Cigarette trajectories were defined as previously described, except there was no biochemical verification.

### Statistical Analysis

Empirical power estimates were assessed by generating multivariate random samples that were matched to expected response patterns for smokers in control and e-cigarette arms with each condition using the same correlation structure of assessments over time as observed in a previous study.^20^ In the e-cigarette condition, we expected larger effects (Cohen *d* = −0.67) on primary outcomes (ie, NNAL) for the approximately 40% of smokers who were able to switch more completely compared with smokers who partially switched (*d* = −0.16). With a median (SD) effect of −0.37 (0.11) across 1000 data sets, simulations revealed that the planned design would provide greater than 82% power for detecting the treatment differences with a sample of 180 participants, with an allowance for up to 20% attrition and α < .05 level.

The primary analysis for all outcomes assessed the differences in the effect of treatment (e-cigarettes) over time by fitting longitudinal models incorporating all measurements for each participant at baseline, week 2 (when available), and week 6. The longitudinal models included treatment, time, and site as fixed effects with an interaction between treatment and time. We explored possible 2- and 3-way interactions of treatment and time with site.

A post hoc exploratory secondary analysis was conducted for all outcomes to assess the differences in the effect of 3 different e-cigarette use trajectories (ie, e-cigarette only, dual use, and combustible cigarettes only) for participants who were randomized to the e-cigarette group. Because individuals could have a different use type at weeks 2 and 6, we fit separate models comparing baseline with week 2 and baseline with week 6, assigning individuals to the type of smoker they were at each time. Each analysis fit a longitudinal model with use type, time, site, and an interaction between use type and time. We explored possible interactions of use type and time with site.

All outcomes were continuous and were log transformed for model fitting since the distributions were highly skewed. We added 1 to all values of any outcome that had at least 1 observed value of 0. Results were not sensitive to choice of the constant added (eTable 2 in Supplement 2). We assumed normally distributed errors with an unstructured covariance matrix. Final models were fitted using restricted maximum likelihood estimation.

Because of the log transformation, changes in outcome (geometric means over time) are interpreted on a relative scale. All relative risks (RRs) are expressed as treatment relative to control. For example, an RR of 0.33 indicates that the risk in the treated group is 0.33 times that in the control group (ie, one-third). This corresponds to a reduction of 67%, or 1.0 − 0.33 = 0.67. Levels at weeks 2 and 6 are reported as a proportion of the baseline level. We included all participants who had at least 1 measurement at baseline, week 2, or week 6 and compared baseline characteristics between participants with missing outcome measurements and participants with complete data. All analyses were adjusted for site because most of the participants with missing data came from San Diego (eTable 3 in Supplement 2). All model assumptions were checked with standard regression diagnostics.

All analyses were performed with R statistical software version 3.6.3 (R Project for Statistical Computing). *P* values were 2-sided, and statistical significance was set at .05. Data were analyzed from September 18, 2019, to September 4, 2020.

## Results

Of 933 participants screened, 126 were randomized to e-cigarette and 61 were randomized to cigarettes as usual; 1 participant was excluded post-randomization, for a final analytic sample of 186 participants , including 92 African American participants and 94 Latinx participants. The mean (SD) age was 43.3 (12.5) years, and 75 (40.3%) were women. Participants smoked a mean (SD) of 12.1 (7.2) cigarettes/d on 6.8 (0.6) d/wk at baseline. At baseline, median (interquartile range) NNAL was 124 (45-197) pg/mL in the e-cigarette group and 88 (58-197) pg/mL in the control group. Additional baseline characteristics are shown in Table 1. Study flow and retention are shown in the Figure. There were no reportable serious adverse events during the study.

**Table 1.** Baseline Characteristics of Study Participants

Variable	Group, No. (%)		
	All (N = 186)	e-Cigarette (n = 125)	Control (n = 61)
Age, mean (SD), y	43.3 (12.5)	44.1 (12.7)	41.7 (11.9)
Women	75 (40.3)	49 (39.2)	26 (42.6)
African American	92 (49.5)	62 (49.6)	30 (49.2)
Latinx	94 (50.5)	63 (50.4)	31 (50.8)
≤High school education	102 (54.8)	68 (54.4)	34 (55.8)
Income ≤200% FPL	138 (75.0)	93 (75.6)	45 (73.8)
Never married	90 (48.4)	59 (47.2)	31 (50.8)
Menthol smoker	102 (54.8)	68 (54.4)	34 (55.7)
Duration smoking, mean (SD), y	16.8 (12.7)	17.5 (12.8)	15.5 (12.5)
Time to first cigarette ≤30 min	135 (72.6)	91 (72.8)	44 (72.1)
Days smoked in past 7 d^a^	6.8 (0.6)	6.9 (0.5)	6.7 (0.9)
Cigarettes/d in past 7 d^a^	12.1 (7.2)	12.4 (7.7)	11.5 (6.1)
Days used e-cigarette in past 7 d^a^	0.05 (0.30)	0.03 (0.28)	0.08 (0.33)
e-Cigarette uses on days used in past 7 d, mean (SD), No.^a^	0.06 (0.45)	0.05 (0.48)	0.09 (0.36)
History of COPD	10 (5.4)	10 (8.1)	0
History of asthma	41 (22.2)	31 (25.0)	10 (16.4)
Any mental health history^b^	107 (58.2)	77 (62.1)	30 (50.0)
History of substance abuse	88 (47.6)	64 (51.2)	24 (40.0)
Biomarkers, median (IQR)			
Urine Cotinine, ng/mL^c^	998 (480-1653)	928 (463-1476)	1061 (534-1720)
Urine NNAL, pg/mL^c^	110 (52-197)	124 (45-197)	88 (58-197)
Carbon monoxide, ppm	17 (11-23)	16 (11-22)	17 (11-25)
Lung function, FEF_25%-75%_, L/s	3.0 (2.1-4.1)	3.0 (2.1-4.1)	2.8 (2.1-4.0)
Respiratory symptoms, No.	10 (5-17)	11 (5-18)	8 (4-13)
Blood pressure, mm Hg			
Systolic	129 (116-142)	130 (115-142)	129 (118-140)
Diastolic	82 (76-89)	81 (76-89)	83 (74-88)

Abbreviations: COPD, chronic obstructive pulmonary disease; FEF_25%-75%_, midexpiratory forced expiratory flow; FPL, federal poverty level; IQR, interquartile range; NNAL, 4-(methylnitrosamino)-1-(3-pyridyl)-1-butanol.

^a^ From 7-day timeline follow-back.

^b^ Self-reported history of depression, anxiety, posttraumatic stress disorder, or schizophrenia.

^c^ Normalized for creatinine.

**Figure.**
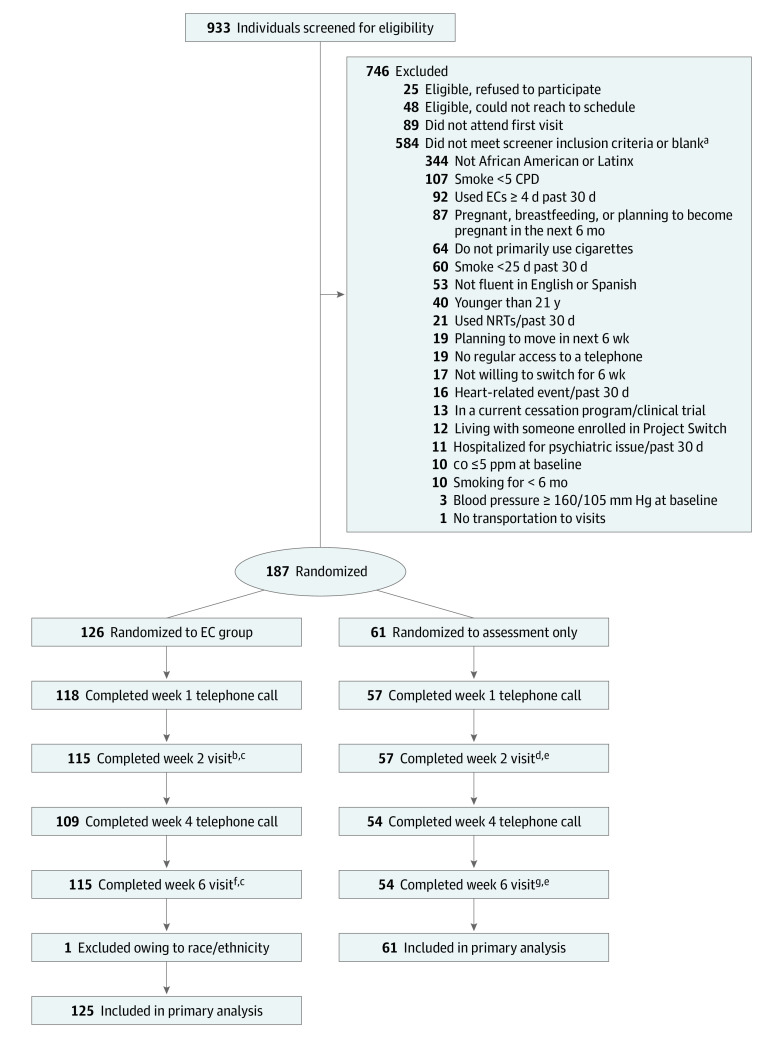
Participant Recruitment Flowchart CO indicates carbon monoxide; CPD, cigarettes per day; EC, electronic cigarette; and NRT, nicotine replacement therapy. ^a^Includes multiple categories. ^b^Four participants missed week 2 but attended week 6. ^c^Seven participants missed week 2 and week 6. ^d^One participant missed week 2 but attended week 6. ^e^Three participants missed week 2 and week 6. ^f^Four participants missed week 6 but attended week 2. ^g^Four participants missed week 6 but attended week 2.

### Missing Data

All participants had at least 1 measurement recorded for all outcome variables, except for 1 participant who missed all NNAL measurements and thus was not analyzed for NNAL. Most missing values occurred among participants at the San Diego site; participants at the San Diego site missing NNAL measurements were similar to those not missing measurements, except that the proportion of never married was higher among the missing (eTable 3 in Supplement 2). Because adjustment for marital status had no effect on results (eTable 4 in Supplement 2), it was not included in final models.

### Within-Group Changes

Compared with baseline, participants in the e-cigarette group reduced several outcomes significantly at week 6, including NNAL (RR, 0.33 [95% CI, 0.26-0.42]), CO (RR, 0.48 [95% CI, 0.41-0.55]), cigarette consumption in the past 7 days among those still smoking (RR, 0.23 [95% CI, 0.18-0.30]), and respiratory symptoms (RR, 0.70 [95% CI, 0.60-0.83]). Cotinine, lung function, and BP did not change significantly between baseline and 6 weeks. There were no changes in any variables from baseline to week 6 within the cigarettes as usual control group (Table 2). Week 2 results were similar to week 6 results (Table 2).

**Table 2.** Effect of e-Cigarettes on Biomarkers of Exposure and Short-term Cardiopulmonary Outcomes

Variable	Within-group change from baseline				Between-group change	
	e-Cigarette (n = 125)		Control (n = 61)			
	RR (95% CI)	*P* value	RR (95% CI)	*P* value	RR (95% CI)	*P* value
NNAL at week 6, pg/mL^a^^,^^b^	0.33 (0.26-0.42)	<.001	0.92 (0.65-1.30)	.64	0.36 (0.23-0.54)	<.001
Cotinine at week 6, ng/mL^a^	0.93 (0.77-1.12)	.45	1.17 (0.90-1.53)	.25	0.80 (0.58-1.10)	.17
Carbon monoxide, ppm						
Week 2	0.45 (0.38-0.52)	<.001	0.98 (0.79-1.22)	.87	0.46 (0.35-0.59)	<.001
Week 6	0.48 (0.41-0.55)	<.001	0.89 (0.73-1.09)	.27	0.53 (0.42-0.68)	<.001
Cigarettes, past 7 d, No.^c^						
Week 2	0.23 (0.18-0.30)	<.001	0.95 (0.73-1.23)	.68	0.25 (0.17-0.36)	<.001
Week 6	0.23 (0.18-0.30)	<.001	0.77 (0.59-1.01)	.06	0.30 (0.20-0.43)	<.001
Respiratory symptoms, per point						
Week 2	0.90 (0.78-1.05)	.18	1.20 (0.97-1.47)	.09	0.75 (0.59-0.97)	.03
Week 6	0.70 (0.60-0.83)	<.001	1.11 (0.87-1.42)	.39	0.63 (0.47-0.85)	.002
Lung function, FEF_25%-75%_, L/s^d^						
Week 2	0.99 (0.92-1.07)	.80	1.01 (0.91-1.11)	.91	0.98 (0.87-1.12)	.81
Week 6	0.96 (0.88-1.04)	.32	1.01 (0.89-1.14)	.93	0.95 (0.82-1.11)	.53
Systolic blood pressure, mm Hg						
Week 2	1.00 (0.98-1.02)	.84	1.01 (0.98-1.04)	.63	0.99 (0.96-1.03)	.78
Week 6	1.01 (0.98-1.03)	.58	1.03 (0.99-1.06)	.14	0.98 (0.94-1.02)	.36
Diastolic blood pressure, mm Hg						
Week 2	0.99 (0.97-1.02)	.64	1.00 (0.97-1.03)	.86	1.00 (0.96-1.04)	.90
Week 6	1.00 (0.98-1.02)	.84	1.00 (0.97-1.03)	.96	1.00 (0.97-1.04)	.87

Abbreviation: FEF_25%-75%_, midexpiratory forced expiratory flow; NNAL, 4-(methylnitrosamino)-1-(3-pyridyl)-1-butanol; RR, relative risk.

^a^ Normalized for creatinine.

^b^ One sample missing.

^c^ From 7-day timeline follow-back among continuing smokers only.

^d^ Mean forced expiratory flow between 25% and 75% of forced vital capacity.

### Between-Group Differences

Compared with the cigarettes as usual group, levels of several outcomes were reduced significantly more in the e-cigarette group at week 6 (Table 2; eFigure 1 in Supplement 2). The e-cigarette group had significantly greater reductions in NNAL (RR, 0.36 [95% CI, 0.23-0.54]; *P* < .001), CO (RR, 0.53 [95% CI, 0.42-0.68]; *P* < .001), respiratory symptoms (RR, 0.63 [95% CI, 0.47-0.85]; *P* = .002), and number of cigarettes smoked in the past 7 days among those still smoking (RR, 0.30 [95% CI, 0.20-0.43]; *P* < .001) than the cigarettes as usual group. Lung function and BP were similar in the 2 groups, and cotinine was not significantly different at week 6. There were no significant differences in treatment effects by site. Results were also comparable when removing 10 participants with a history of chronic obstructive pulmonary disease (eTable 5 in Supplement 2). Week 2 results were similar to those at week 6 (eTable 6 in Supplement 2).

### Change by e-Cigarette Use Trajectory

At week 6, approximately one-quarter of participants in the e-cigarette group (32 participants [28.1%]) were classified as exclusive e-cigarette users (verified with CO <6 ppm), more than half (66 participants [57.9%]) were dual users, and a small number were exclusive cigarette smokers (16 participants [14.0%]) (Table 3). At 6 months, 23 participants (24.0%) were still classified as exclusive e-cigarette users (non-bioverified), 32 participants (33.3%) were dual users, 31 participants (32.3%) were exclusive cigarette smokers, and 10 participants (10.4%) did not use either e-cigarettes or cigarettes.

**Table 3.** e-Cigarettes Group Switching Patterns

Nicotine use	Participants, No. (%)		
	Week 2 (n = 114)^a^	Week 6 (n = 114)^a^	Month 6 (n = 96)^b^
Exclusively e-cigarettes^c^	32 (28.1)	32 (28.1)	23 (24.0)^d^
Dual e-cigarettes and cigarettes	77 (67.5)	66 (57.9)	32 (33.3)^e^
Cigarettes and no e-cigarettes	4 (3.5)	16 (14.0)	31 (32.3)^f^
No e-cigarettes or cigarettes	1 (0.9)	0	10 (10.4)^g^

^a^ Includes those in e-cigarette group in the final analytic sample who completed study visit. Tobacco use classification using 7-day timeline follow-back.

^b^ Eighteen participants who completed the study could not be reached for the month 6 telephone call. Tobacco use classification using past 30-day cigarette and e-cigarette use.

^c^ Exclusive e-cigarettes bioverified with carbon monoxide less than 6 ppm for week 2 and week 6 but not month 6.

^d^ Of 23 participants, 15 (65%) were exclusive e-cigarette users at week 6 and 8 (35%) were dual users at week 6.

^e^ Of 32 participants, 23 (71.9%) were dual users at week 6, 5 (15.6%) were cigarettes-only at week 6, and 4 (12.5%) were exclusive e-cigarette users at week 6.

^f^ Of 31 participants, 18 (58.0%) were dual users at week 6, 7 (22.6%) were cigarettes-only at week 6, and 6 (19.4%) were exclusive e-cigarette at week 6.

^g^ Of 10 participants, 4 (40%) were exclusive e-cigarette users at week 6 and 6 (60%) were dual users at week 6.

Participants who switched exclusively to e-cigarettes demonstrated significant reductions from baseline in NNAL (RR, 0.08 [95% CI, 0.05-0.13]), CO (RR, 0.20 [95% CI, 0.16-0.24]) and self-reported respiratory symptoms (RR, 0.58 [95% CI, 0.42-0.81]) at week 6 (Table 4; eFigure 2 in Supplement 2). Participants classified as dual users also experienced significant reductions in NNAL (RR, 0.49 [95% CI, 0.36-0.66]), CO (RR, 0.60 [95% CI, 0.52-0.69]), and self-reported respiratory symptoms (RR, 0.69 [95% CI, 0.55-0.87]) at week 6 (eTable 7 in Supplement 2).

**Table 4.** Biomarkers of Exposure and Short-term Cardiopulmonary Outcomes by e-Cigarette Group Trajectory

Variable	Relative risk (95% CI)					
	Within group change from baseline			Between group change		
	e-Cigarette only	Dual use	Cigarette only	e-Cigarette vs cigarette	Dual use vs cigarette	e-Cigarette vs dual use
NNAL at week 6, pg/mL^a^	0.08 (0.05-0.13)	0.49 (0.36-0.66)	0.96 (0.51-1.82)	0.08 (0.04-0.18)	0.51 (0.25-1.02)	0.17 (0.10-0.28)
Cotinine at week 6, ng/mL^a^	1.01 (0.71-1.44)	0.88 (0.69-1.12)	1.09 (0.65-1.82)	0.93 (0.50-1.72)	0.81 (0.46-1.42)	1.15 (0.75-1.77)
Carbon monoxide, ppm						
Week 2	0.20 (0.15-0.26)	0.60 (0.50-0.71)	0.67 (0.31-1.42)	0.30 (0.14-0.67)	0.90 (0.41-1.94)	0.34 (0.25-0.46)
Week 6	0.20 (0.16-0.24)	0.60 (0.52-0.69)	1.05 (0.78-1.40)	0.19 (0.13-0.27)	0.58 (0.42-0.80)	0.33 (0.26-0.42)
Respiratory symptoms, per point						
Week 2	0.80 (0.62-1.04)	0.87 (0.73-1.03)	1.45 (0.70-2.98)	0.55 (0.26-1.19)	0.60 (0.29-1.26)	0.92 (0.68-1.26)
Week 6	0.58 (0.42-0.81)	0.69 (0.55-0.87)	0.96 (0.59-1.55)	0.61 (0.34-1.09)	0.72 (0.42-1.22)	0.84 (0.56-1.26)
Lung function, FEF_25%-75%_, L/s						
Week 2	0.93 (0.81-1.07)	1.05 (0.96-1.15)	0.67 (0.45-0.98)	1.40 (0.93-2.11)	1.57 (1.06-2.34)	0.89 (0.75-1.05)
Week 6	1.00 (0.85-1.17)	0.93 (0.83-1.04)	0.98 (0.78-1.24)	1.01 (0.76-1.34)	0.95 (0.73-1.22)	1.07 (0.88-1.31)
Systolic blood pressure, mm Hg						
Week 2	1.00 (0.96-1.05)	1.00 (0.97-1.03)	1.01 (0.90-1.14)	0.99 (0.87-1.12)	0.99 (0.87-1.12)	1.00 (0.95-1.05)
Week 6	1.02 (0.97-1.06)	1.00 (0.97-1.03)	1.01 (0.95-1.07)	1.01 (0.93-1.09)	0.99 (0.93-1.06)	1.01 (0.96-1.07)
Diastolic blood pressure, mm Hg						
Week 2	1.01 (0.97-1.05)	0.99 (0.96-1.02)	1.00 (0.88-1.13)	1.01 (0.89-1.15)	0.99 (0.88-1.12)	1.02 (0.97-1.07)
Week 6	1.00 (0.96-1.04)	1.00 (0.97-1.03)	1.02 (0.96-1.07)	0.99 (0.92-1.06)	0.98 (0.92-1.04)	1.00 (0.96-1.05)

Abbreviations: FEF_25%-75%_, midexpiratory forced expiratory flow; NNAL, 4-(methylnitrosamino)-1-(3-pyridyl)-1-butanol.

^a^ Normalized for creatinine.

The e-cigarette trajectory subgroups differed significantly for NNAL and CO levels. Exclusive e-cigarette users had the most pronounced changes, followed by dual users, and then exclusive cigarette smokers (Table 4). At week 6, exclusive e-cigarette users had significantly greater reductions in NNAL and CO levels than dual users and cigarette smokers. Dual users also had greater reduction of CO levels compared with exclusive cigarette smokers. Week 2 results were generally similar, with differences mainly arising because of imprecision arising from the small number of exclusive cigarette smokers at week 2 (4 participants [3.5%]). There were no significant differences in treatment effects by site.

## Discussion

In this randomized clinical trial comparing an NSPS e-cigarette with cigarette smoking, a significant reduction in the primary outcome, NNAL, was observed in e-cigarette users. e-Cigarette users also experienced a significant reduction in CO and self-reported respiratory symptoms. Cotinine was stable over time and did not vary between e-cigarette and cigarettes as usual groups, reflecting adequate nicotine replacement by e-cigarette. Additionally, the lack of between-group change in cotinine and BP suggests there was no increased risk from greater exposure to nicotine. There were no interactions by site for any outcomes, reflecting consistency in effects across populations.

Reductions in NNAL and co, and improvements in respiratory symptoms were particularly pronounced among participants who switched exclusively to e-cigarettes, which is consistent with studies of other e-cigarettes.^20,26,27,28,29,30^ Bioverified exclusive switching was maintained from week 2 to week 6, and approximately one-quarter of participants self-reported exclusive e-cigarette use at 6 months. Dual use of e-cigarettes and cigarettes was the most common tobacco use trajectory, as has been reported by Piper et al.^31,32^ Participants in the e-cigarette group who continued smoking while also using e-cigarettes significantly reduced their cigarette consumption from baseline to week 6 while maintaining cotinine levels, indicating that their primary source of nicotine was e-cigarettes. Although caution is needed given the observational subanalysis, results suggest that dual use of e-cigarettes and cigarettes did not create an additive burden on biomarkers of toxicant exposure compared with cigarette smoking in this 6-week trial.

### Limitations

This study has some limitations. The 6-week study period was insufficient to understand the effects of e-cigarettes over a sustained time, and longer-term studies are needed. Certain effects, particularly lung function, are unlikely to be detected in a 6-week period. Furthermore, expansion to additional cardiopulmonary measures and e-cigarette-specific measures (eg, metals, acrolein) are recommended in future research to increase our understanding of the impact of e-cigarettes on the cardiopulmonary system.^26,33^

Enrollment of African American participants was limited to Kansas City, Missouri, and Latinx participants to San Diego, California. While there were differences in results by site, generalizability would be improved by a more comprehensive sampling strategy. Additionally, generalizability is limited by the provision of e-cigarettes during the 6-week trial, although results of the 6-month follow-up survey suggest that most participants continued using e-cigarettes, which required acquiring their own pods. Only 1 NSPS e-cigarette was tested, so results may not be generalizable to other types or brands of these devices or to older, non-NSPS devices. Participants were primarily light smokers, which is an understudied but increasing group of smokers who experience significant tobacco-related morbidity and mortality.^34,35^ However, their cigarette consumption was lower than the national mean for all smokers; therefore, results may not generalize to heavier smokers.

## Conclusions

This randomized clinical trial found that the use of NSPS e-cigarettes for replacing cigarettes led to significant reduction in a primary pulmonary carcinogen, NNAL, for African American and Latinx smokers. There were also significant reductions in cigarettes smoked per day, co, and respiratory symptoms and no increase in nicotine exposure. Furthermore, about one-fourth of participants randomized to e-cigarettes were able to fully cease use of combustible cigarettes at week 6. Reduction in cigarettes and biomarkers of exposure in this study suggest potential of NSPS e-cigarettes as a harm reduction strategy for members of the 2 largest minority groups in the US who face significant health disparities.

## Notes

Supplement 1. **Trial Protocol**

Supplement 2. **eTable 1.** Pod Flavor Selection of e-Cigarette Group Participants at Baseline and Week 2 **eTable 2.** Sensitivity Analysis for the Addition of a Constant to NNAL Measurements at Week 6 to Enable a Logarithmic Transformation in the Presence of Some Zero Measurements **eTable 3.** Baseline Characteristics According to Whether Participants Had a 6-Week NNAL Measurement **eTable 4.** Effect of e-Cigarettes on Biomarkers of Exposure and Short-Term Cardiopulmonary Outcomes Adjusted for Marital Status **eTable 5.** Sensitivity Analysis Removing the People With a History of Chronic Obstructive Pulmonary Disease for Effect of e-Cigarettes on Biomarkers of Exposure and Short-Term Cardiopulmonary Outcomes **eTable 6.** Medians of Outcomes at Baseline, Week 2 and Week 6 by Study Randomization Group **eTable 7.** Medians of Outcomes at Baseline, Week 2 and Week 6 by Week Six Tobacco Use Trajectory **eFigure 1.** Patterns of Change in NNAL, co, and Cotinine for e-Cigarette vs Control Group **eFigure 2.** Patterns of Change in NNAL, co, and Cotinine for e-Cigarette Trajectory Groups

Supplement 3. **Data Sharing Statement**
