# Supplementary material for: Effect of Pod e-Cigarettes vs Cigarettes on Carcinogen Exposure Among African American and Latinx Smokers: A Randomized Clinical Trial
Source: JAMA Netw Open. 2020 Nov 18;3(11):e2026324. doi: 10.1001/jamanetworkopen.2020.26324 (PMC7675102; doi:10.1001/jamanetworkopen.2020.26324)
Supplement: Supplement 1. — Trial Protocol [file jamanetwopen-e2026324-s001.pdf]

TOXICANT EXPOSURE AND HARM PERCEPTIONS IN CIGARETTE SMOKERS WHO USE OR DO NOT USE E-CIGARETTES

STUDY PROTOCOLS & PROCEDURES

I. Introduction

II. Study Sites

III. Study Personnel

IV. Participant Recruitment

V. Participant Screening and Enrollment

VI. Study Design and Timeline

VII. Participant Retention

VIII. Laboratory Procedures and Sampling

IX. Laboratory Analysis

X. Study Visits Overview

XI. Study Questionnaires

XII. Data Management

XIII. Recording and Reporting of Adverse Effects

I. Introduction

This study, **Toxicant Exposure and Harm Perceptions in Cigarette Smokers Who Use or Do Not Use E- Cigarettes** is funded by the National Institutes of Health and registered on ClinicalTrials.gov.

Document Purpose

This document is intended to detail all relevant study protocols and procedures. Given rapid advancement in the understanding of the effects of smokers switching to electronic cigarettes, particularly on toxicant exposure (our primary outcome), we considered how to maximize the scientific contribution of the project. The body of work on the use of electronic cigarettes for risk reduction has been conducted with the general population and is now moving into vulnerable populations who carry a substantial disease burden from smoking. The investigative team is uniquely positioned to create progress in the field by examining the research questions with ethnically diverse populations who face significant barriers to smoking cessation. A commitment was made at the San Diego site to enroll Latinx smokers and to offer the study in Spanish in order to address the research questions with an under-studied population. NIH prior approval and Institutional Review Board (IRB) approval was granted to add the University of Kansas Medical Center as a study site to enroll African American smokers. This change enhances the ethnic diversity of enrolled participants as well as expands the science on risk reduction. The second study site (University of Kansas Medical Center) agreed to rely on the IRB of the primary institution (CSUSM).

Additionally, because this project was funded on the first round, reviewer comments were addressed in the implementation of the study protocol post-proposal. These changes impacted inclusion/exclusion criteria and the length of the intervention period. There were no changes to aims, hypotheses, or sample size.

The following Project Summary reflects our implemented protocol and contains an updated premise reflecting the ethnic minority population focus.

Project Summary

This proposal addresses a critical gap in the research regarding the use of electronic cigarettes (ECs) for harm reduction by ethnic minority smokers. Smokers cite reducing perceived harm from smoking as a leading reason for using ECs, and ECs have been suggested by tobacco researchers as a potential harm reduction vehicle for smokers who cannot or will not achieve smoking cessation. The National Academies of Science concluded that ECs pose significantly less exposure to toxicants and less short-term health risks than combustible cigarettes. However, smokers with less education and from ethnic minority backgrounds are less likely to switch to exclusive EC use. Socioeconomic disparities in switching to ECs will perpetuate a greater burden of tobacco-related death and disease among disadvantaged populations. As a human behavioral EC study in which toxicant exposure via the tobacco-specific nitrosamine, NNAL, a highly potent pulmonary carcinogen will be measured, this project addresses a

critical barrier in the field and advances science on the widely used practice of EC use for harm reduction among the two largest ethnic minority groups in the US, Latinx and African Americans. Furthermore, this will be among the first studies to determine how uptake of ECs affects cognitive-perceptual factors involved in sustained tobacco use, such as risk perceptions, utility of smoking, self-efficacy to quit, and tobacco dependence among under-represented minority smokers. This study is directly relevant to the NIH's mission to address cancer risk factors and supports the long-term objective to reduce the disease burden of tobacco use. In the current proposal, Latinx (n=90) and African American (n=90) cigarette smokers will be randomized in a 2:1 fashion to an EC group (n=60 per racial/ethnic group; N=120) or an assessment-only control group (n=30 per racial/ethnic group; N=60). Those randomized to EC will receive a 6-week supply of a fourth generation EC starter kit with their choice of liquid flavor from a standard list. Those randomized to assessment only (n=60) will not be provided with an EC. Tobacco consumption in both groups will be assessed at weeks 0 (baseline), 2, and 6. Changes in toxic exposure (NNAL, a primary lung carcinogen and the primary outcome) and carbon monoxide (CO) will be measured from baseline to week 6. Planned covariates include study site, gender, income, tobacco dependence score, and mental health symptoms.

Aim 1. To characterize the toxic exposure of cigarette smokers randomized to the EC group compared to cigarette smokers randomized to assessment-only controls

Hypothesis 1: It is hypothesized that toxicant exposure from baseline to week 6 will be significantly lower in the EC group compared to the assessment-only group. *Hypothesis 2:* It is hypothesized that change in cigarette consumption in the EC group from baseline to week 6 will be associated with reduction in toxicant exposure from baseline to week 6.

Aim 2. To assess the effects of uptake of e-cigarettes on perceptions of harm and utility of products

It is hypothesized that from baseline to week 6, the EC as compared to control arm a) will have decreased perceptions of harm to their health from current tobacco use, b) will increase positive utility of EC and increase negative utility of cigarette products, and c) will have decreased expectations of the difficulty of quitting cigarette smoking.

Aim 3. To understand patterns of tobacco product consumption among smokers switching to electronic cigarettes

Changes in tobacco product consumption (cotinine-verified), subjective effects of smoking, and levels of tobacco dependence from baseline to week 6 will be examined in the EC group and baseline predictors of those patterns, including demographic, smoking history, and psychosocial characteristics will be identified.

This project will advance science on the widely used practice of EC use for harm reduction by examining change in toxicant exposure via NNAL and change in cognitive-perceptual variables among African American and Latinx smokers switching to EC compared to smokers who continue to use cigarettes alone. Study findings will have major public health implications, particularly for smokers who have experienced difficulty

in quitting cigarettes and for whom EC use as a harm reduction strategy has been considered. The study will enhance the research environment at the PI's university which is classified as both a Hispanic Serving Institution and an Asian American-Native American-Pacific Islander Serving Institution.

Important Terms and Abbreviations

NNAL	4-(methylnitrosamino)-1-(3-pyridyl)-1-butanol
REDCap	Research Electronic Data Capture
CO	Carbon Monoxide
TLFB	Timeline Followback

II. Study Sites

1. San Diego
    a. California State University San Marcos – 333 S Twin Oaks Valley Rd, San Marcos, CA 92096
    b. San Diego State Research Foundation – 4283 El Cajon Blvd Suite 226, San Diego, CA 92105
    c. Neighborhood Healthcare, Behavioral Health – 425 N. Date Street Escondido, CA 92025
2. Kansas City – Swope Medical Center – 3801 Blue Pkwy, Kansas City, MO 64130

III. Study Personnel

Name	Organization	Role
Kim Pulvers, PhD, MPH	California State University San Marcos	Principal Investigator
Jasjit Ahluwalia, MD, MPH, MS	Brown University	Co-Investigator
Nicole Nollen, PhD	University of Kansas School of Medicine	Co-Investigator
Tricia Snow, MA	University of Kansas Medical Center	Project Manager
Brian Hernandez, BA	University of Kansas Medical Center	Lead Researcher
Michael Arnold, BA	University of Kansas Medical Center	Research Assistant
Myra Rice, BA	California State University San Marcos	Project Manager
Amanda Dean, BA	California State University San Marcos	Research Assistant
Dalia Hipolito, BA	California State University San Marcos	Research Assistant
Jennifer Mosley, BA	California State University San Marcos	Research Assistant
Mirella Orozco, BA	California State University San Marcos	Research Assistant
Ana Leon, BA	California State University San Marcos	Research Assistant
Justin Sanchez	California State University San Marcos	Research Assistant
Crystal Marez, BA	California State University San Marcos	Research Assistant
Juan Alva	California State University San Marcos	Research Assistant
John Le	California State University San Marcos	Research Assistant
Laura Wells, BA	California State University San Marcos	Research Assistant
Jeremy Mills-Shimmel	California State University San Marcos	Research Assistant
Shyla Everett	California State University San Marcos	Research Assistant
Daniell Derry	California State University San Marcos	Research Assistant
Flavia Ponce, BS	University of California San Diego	Research Assistant
Nathan Au-Yeng, BS	University of California San Diego	Research Assistant
Madison Garrett	University of California San Diego	Research Assistant
Alexis Osuna	San Diego State University	Research Assistant

Table 1. Study personnel for Project Switch.

IV. Participant Recruitment

Recruitment will utilize online sources including Craigslist and Facebook, as well as radio advertisements, newspaper advertisements, flyers, information cards, clinic referrals, participant referrals, and community outreach. Enrolled participants will be encouraged to refer other smokers to the study and provided material such as information cards, pens, and t-shirts to facilitate referrals.

Sample Size. We estimated our sample requirements using power analyses for primary aims to characterize the reduction in toxic exposure of cigarette smokers randomized to the e-cigarette group compared to cigarette smokers randomized to assessment-only controls (Aim 1:H1) from baseline to week 6 will be significantly lower in the EC group compared to the assessment-only group. Empirical power estimates were assessed by generating multivariate random samples that were matched to the expected response patterns for smokers in control and switching arms with each condition using the same correlation structure of assessments over time as observed in a previous study. In the switching condition we expect larger effects (d=-0.67) on primary outcomes (NNAL) for the ~40% of smokers able to switch more completely relative to smokers partially switching (d=-0.16). With no change expected in control, we powered primary outcomes for end-of-treatment analyses using an average of effects given 30% of switching smokers with large and 70% with small effects. With a median effect of -0.37 (SD=0.11) across 1000 data sets, simulations revealed that the planned design would provide greater than 0.82 power for detecting the treatment differences with a sample of 180 subjects, with an allowance for up to 20% attrition. For Aim1: H2 we will have power to detect moderate effects of changes in cigarette consumption on change in toxicants. In published effects of differences in consumption levels on nitrosamines, 77 differences in consumption (20 vs 10 cig/day) reflected mean difference in log NNAL of 2.60-2.35=0.24 and effect sizes with d=0.48 after 10 weeks 78. We estimate adequate power >0.80 given expected cigarette reductions. Changes in cognitive measures (Aim 2) including decreased perception of harm, average differences in utility of combustible and EC, and increased self-efficacy for quitting are expected to be moderately associated with EC use. Empirical power analyses using regression models of change in cognitive measures over 1000 simulated data sets support the ability to detect moderate effects (d>0.30) using a standardized difference in means of cognitive measures with power >0.80.

V. Participant Screening and Enrollment

Prospective participants will be determined eligible or ineligible within 48 hours of initial contact. If eligible, the baseline visit will be scheduled, and a postcard reminder sent out. Screening will occur over the phone, in person, or with an online screening survey developed in REDCap.

Final eligibility is not decided until baseline visit, contingent on blood pressure under 160 (systolic) and 105 (diastolic), carbon monoxide over 5 PPM to validate smoking status, and researcher-assessed stability. Participants who do not meet criteria will not be included in the study and given a smoking cessation referral. San Diego ineligible participants will be referred to the California Smokers' Helpline and Kansas City ineligible participants will be referred to the Kansas Tobacco Quitline. Transportation will be provided to and from study visits when needed in San Diego.

A. Inclusion Criteria

- ≥ 21 years of age
- Smoked cigarettes on ≥ 25 of past 30 days
- Smoked ≥ 5 cigarettes per day on days that smoked
- Smoked cigarettes for ≥ 6 months
- Carbon monoxide > 5 PPM at baseline
- Blood pressure systolic or diastolic < 160/105
- Hispanic/Latinx at San Diego or Black/African American at Kansas City
- Fluent in English or Spanish
- Willing to switch from smoking cigarettes to e-cigarettes for 6 weeks
- Regular access to a telephone
- Transportation to attend Swope Health Central in next six weeks (Kansas City)

B. Exclusion Criteria

- Primary use of other tobacco products or equal use of cigarettes and other tobacco products
- E-cigarette use on ≥ 4 of the past 30 days
- Currently in a smoking cessation program or other clinical trial
- Use of nicotine replacement therapy or medicine which aids smoking cessation in the past 30 days
- Hospitalizations for a psychiatric issue in the past 30 days
- Heart-related event in the past 30 days. Examples include heart attack, stroke, severe angina (i.e. chest pain), ischemic heart disease, and vascular disease
- Planning to move out of San Diego or Kansas City in the next 6 weeks
- Another person in the household enrolled in the study
- Women: pregnant, breastfeeding, or planning to become pregnant in the next six months
- Screener judgment about unstable mental status or health status

VI. Study Design and Timeline

This study is a randomized controlled trial using a 2:1 study randomization ratio. The treatment group will receive an e-cigarette and nicotine pods for six weeks and are encouraged to make a complete switch from combustible cigarettes to e-cigarettes.
The study will consist of three in-person visits (baseline, week 2, and week 6) in which measurements are conducted and behavioral support is provided to the e-cigarette group. Phone calls will be scheduled between the visits (week 1 and week 4) to confirm appointments, collect data on tobacco use, and to support switching to e-cigarettes (e-cigarette group only).

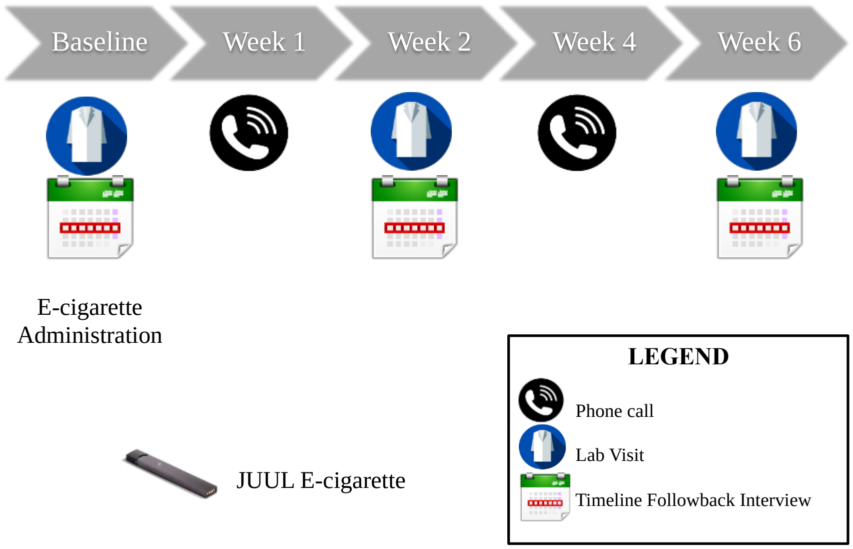

Figure 1. Study Timeline.

Informed Consent. The informed consent will be reviewed by having the researcher read the consent form out loud. The participant will be given time to read the informed consent form and ask questions. It will be made clear in the consent form that the participant has the option to take more time to consider their desire to participate in the study and reschedule at a later date, or to decline to participate at any point. The study begins once the participant signs the consent form, indicating understanding of the procedures, requirements, and consent.

Incentives. Participants will be compensated for their time during the lab visits at escalating increments of $20 at baseline, $40 at week 2, and $60 at week 6, totaling up to $120. Participants in the e-cigarette group are instructed that full compensation is contingent upon bringing back their used and unused pods to their next visit. At week 2 and week 6, $20 of compensation is contingent upon bringing back pods. Those in the e-cigarette group have the opportunity to participate in a follow-up phone assessment six months after enrollment, entailing an additional $20 compensation.

Study E-cigarettes. Participants in the e-cigarette group will be provided JUUL, a nicotine-salt based e-cigarette (see Figure 2). Participants will have the flavor selection of Virginia tobacco, cool mint, menthol, or mango pods containing .07 mL nicotine (5% nicotine). Pod use will be tracked using the Pod Count Form, requiring participants to bring to lab visits all used, unused, and partially used pods. Pods will be distributed based on cigarette use, and determined using the Pod Count Calculation Form. Education about switching will be verbally provided to the e-cigarette group. Participants will engage in motivational enhancement-based action planning and will receive JUUL usage instructions. Participants in the e-cigarette group will be provided a compatible wall adapter to ensure proper charging, a device carrying case, Q-tips to clean the device as needed, and a gallon Ziplock bag to store used pods.

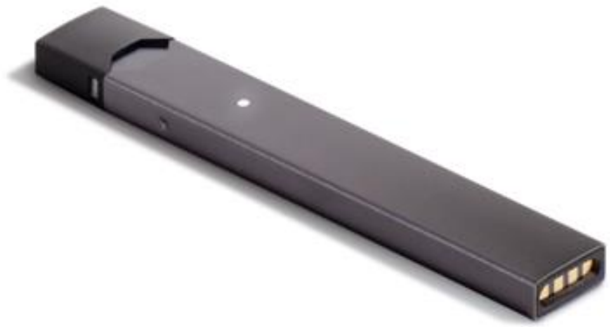

Figure 2. JUUL electronic cigarette. Reprinted from *JUUL Labs, Inc,* Retrieved December 9, 2018, from www.juul.com. Copyright 2018.

VII. Participant Retention

Participant retention will be maintained by using a variety of contact methods including call, text, email, physical letter in the mail, and reaching out to emergency contacts. No more than six contact attempts, of various forms, will be made. Contacting emergency contacts and sending a letter will be the last efforts made to contact an unreachable participant. Additionally, participants will be sent postcards reminding them of the day and time of their next lab visit, and appointment reminder cards will be given out at the baseline and week 2 visit reminding about upcoming phone calls.

Lab visits will have pre-determined time windows. The week 2 visit can be scheduled up to one week before or after the originally scheduled date of two weeks after the baseline visit. The week 6 visit can be scheduled up to four weeks before or after the originally scheduled date of six weeks after the baseline visit. Ripple protocol details the scheduling protocol.

VIII. Laboratory Procedures and Sampling

Data from biological measurements and samples will be recorded on paper and/or in REDCap.

The table below shows the equipment used for the biological measures, and timepoints taken.

Measure	Equipment	Baseline	Week 2	Week 6
Carbon Monoxide	coVita|Bedfont Micro+ Smokerlyzer®	X	X	X
Systolic Blood Pressure, Diastolic Blood Pressure, and Pulse	Omron® BP742N 5 Series Upper Arm Blood Pressure Monitor	X	X	X
Height	Health o meter® 500KL	X		

	Professional Digital Scale			
Weight	Health o meter® 500KL Professional Digital Scale	X		X
Spirometry: FVC, FEV1, FEV1/FVC%, PEF, and FEF25-75%	Futuremed® Discovery-2™ Desktop Spirometer	X	X	X
Saliva (10 San Diego participants)	Saliva collection aid (straw): SB-WS No.61/524,096<br><br>Collection tube: Wheaton 1.8 mL yellow cap collection tube	X		X
Nasal Swab (20 Kansas City Participants)	Leukosorb tubes, Q-tips, saline	X		X
Urine	Dynarex Specimen Container 4oz. 118cc reorder No. 4253<br><br>Aliquots: Wheaton 20 ml disposable scintillation vials	X	X	X

Table 2. Biological measures, timepoints, and equipment for Project Switch.

CO measurement. Exhaled breath samples will be taken at all visits for carbon monoxide (CO), a by-product of smoke, using a Bedfont Micro+ Smokerlyzer. At baseline, CO measurement will be taken to confirm eligibility. A carbon monoxide level over 5ppm verifies smoking status. A carbon monoxide level 5ppm or lower at baseline makes a person ineligible to participate in the study. Participants will be allowed the opportunity to re-screen two weeks later.

Blood pressure. Systolic and diastolic blood pressure will be measured for screening purposes and throughout the study using a digital blood pressure cuff. If, at the baseline or week 2 visit, a participant's systolic blood pressure is greater than or equal to 160 mm Hg or their diastolic blood pressure is greater than or equal to 105 mm Hg, they will not be eligible to participate or continue participating in the study. Pulse will also be measured but not as an exclusion criteria. Participants who are ineligible at baseline due to uncontrolled blood pressure will be allowed the opportunity to re-screen two weeks later.

Height and weight. A medical scale will be used for a one-time measure of height. Weight will be measured at baseline and week 6 visit, with shoes removed.

Spirometry. Lung function will be measured at all lab visits using a Discovery-2 SpiroVision spirometer. At each session, participants will complete a minimum of three

maneuvers (depending on the quality) consisting of a strong exhale and a strong inhale. Participants will be given a mouthpiece at baseline that will be stored and used at subsequent visits. Participants will be given the option to manually hold their nose closed or to apply a nose clip.

Spirometer measures include: (1) forced vital capacity, FVC (2) Forced expiratory volume in one second, FEV1 (3) The percentage of the FVC expired in one second, FEV1/FVC% (4) peak expiratory flow, PEF (5) Forced expiratory flow over the middle one half of the FVC, FEF25-75%.

Saliva sampling. Saliva sampling is an exploratory addition to this study and will be taken from ten San Diego participants at baseline and week 6. The decision to take saliva sampling in San Diego resulted in an increase from the original n=90 to an n=94. Samples will be frozen and transported the testing facility on dry ice.

Nasal swab sampling. Nasal swab sampling is an exploratory addition to this study and will be taken from twenty Kansas City participants at baseline and week 6. The decision to take nasal swab sampling in Kansas City resulted in an increase of two participants for a final n=92. Samples will be frozen and transported the testing facility on dry ice.

Urine sampling. Urine will be collected at all lab visits and refrigerated until aliquoting. Participants will be instructed to provide at least half of the specimen cup of urine (about 60mL). Participants will be offered water at the beginning of lab sessions to help facilitate sampling.

Urine will be processed by aliquoting the sample into two smaller samples, each 15 mL. One sample will test for cotinine and need to be pre-treated with sodium bisulfate to obtain a pH between 2 and 3. The second sample will test for NNAL and will receive no additional treatment. Both samples will be frozen and stored at -20 °C before shipping with dry ice for testing.

A urine log will provide the following information for each urine sample: (1) participant ID (2) study time point (3) date: mm/dd/yyyy (4) testing for: NNAL or cotinine. Each lab visit will produce two samples stored for further testing.

Although urine will be taken at all three lab visits, only baseline and week 6 urine will be sent for testing. In the case of missing week 6 samples, week 2 samples will be sent instead as a method of imputation.

Sample shipment. Specimens will be transported in a leak-proof Styrofoam container in leak-proof secondary packaging, with absorbent material placed at the bottom and sides of the container for absorption. 10-15lbs dry ice will be used to maintain storage temperature during transport.

IX. Laboratory Analysis

Urine: cotinine ng/mL, creatinine mg/mL, nitrosamine 4-(methylnitrosamino)-1-(3-pyridyl)-1-butanol (NNAL) pg/mL, and 3-hydroxycotinine ng/mL

Saliva: Cytokine Panel and C-Reactive Protein

Nasal Swab: MMP9 ELISA in pg/mL and TGF-B1 ELISA in pg/mL

X. Study Visits Overview

Baseline	Week 1	Week 2	Week 4	Week 6
Participant consent	Follow-up check-in	Urine test	Follow-up check-in	Saliva sample (San Diego)
Saliva sample (San Diego)	Yesterday tobacco use	Blood pressure test (must be less than 160/105)	Yesterday tobacco use	Urine test
Complete contact sheet		Carbon monoxide test		Blood pressure test
Blood pressure test (must be less than 160/105)		Eligibility check		Carbon monoxide test
Carbon monoxide test (must be greater than 5 ppm)		Follow-up check in sheet (EC group)		Nasal swab (Kansas City)
Nasal swab (Kansas City)		Timeline follow back		Weight
Final eligibility check and Study ID assignment		Spirometry test		Follow-up check in sheet (EC group)
Randomization		Week 2 survey		Timeline follow back
Charge JUUL (EC group)		Collect used JUUL pods (EC group)		Spirometry test
Timeline follow back		Provide more JUUL pods (EC group)		Week 6 survey
Pod count form (EC group)		Schedule visits/phone calls		Collect used JUUL pods (EC group)
Height and weight		Compensation		Tobacco cessation referral
Baseline survey part 1				Exit survey
Spirometry test				Compensation
Baseline survey part 2				
Cigarette questions and breathing conditions				
E-cigarette switching fact sheet (EC group)				
E-cigarette use instructions (EC group)				
E-cigarette trial and				

subjective effects questionnaire (EC group)				
Baseline action planning (EC group)				
Schedule visits/phone calls				
Reimbursement schedule				
Urine test				
Compensation				

Table 3. Surveys and tests by study timepoint.

At each lab visit, participants will receive compensation and sign a receipt. At the end of the study, participants will be given a referral to the California Smokers' Helpline (1-800-NO-BUTTS) or Kansas Tobacco Quitline (1-800-QUIT-NOW).

XI. Study Questionnaires

Surveys will contain items on demographics, smoking measures, tobacco measures, physiological measures, and psychosocial assessments. Given the possibility of low literacy among participants, surveys will be verbally administered along with visual cue cards.

XII. Data Management

Missing data will be minimized through extensive training of research staff in addition to follow-up with participants. Data will be entered into REDcap electronically and paper forms (biological measures and pod count forms) will be stored in participant files. Paper forms with identifiable information such as consent and contact information will be stored in a separate, locked location. TLFB data will be entered into excel, with paper calendars and data entry forms stored in participant files. REDcap entry will happen after the lab session and will be audited and checked by project managers daily. REDCap is a secure, HIPAA-compliant, web-based application designed to support data capture for research studies. All protocol-specified data will be linked by unique subject ID, assigned at randomization. Paper files will be stored on site in a locked filling cabinet and any forms with identifying information (consent form and contact sheet) will be kept in a separate location. Data is only accessible to personnel involved with this research. Access is limited by the research facility being locked at all times.

XIII. Recording and Reporting of Adverse Effects

Development of any adverse effects will be monitored closely by a project manager such that fields capturing adverse events will be reviewed daily. At each study visit, participants will be asked about the development of any other, new, or worsening symptoms. At each visit and each phone call after the initial visit, participants in the e-cigarette condition will be asked if they were experiencing any barriers to switching to e-cigarettes or any concerns about switching. This open-ended format is intended to capture any unexpected adverse events. Unexpected serious adverse events will be reported to the CSUSM IRB and assessed by the PI to determine if related to e-

cigarette use, and if so, reported to the NIH and FDA. Participants will be encouraged to contact staff if they have any questions, problems using their e-cigarette, or if any adverse events develop.
