# Supplementary material for: Effect of Pod e-Cigarettes vs Cigarettes on Carcinogen Exposure Among African American and Latinx Smokers: A Randomized Clinical Trial
Source: JAMA Netw Open. 2020 Nov 18;3(11):e2026324. doi: 10.1001/jamanetworkopen.2020.26324 (PMC7675102; doi:10.1001/jamanetworkopen.2020.26324)
Supplement: Supplement 2. — eTable 1. Pod Flavor Selection of e-Cigarette Group Participants at Baseline and Week 2 eTable 2. Sensitivity Analysis for the Addition of a Constant to NNAL Measurements at Week 6 to Enable a Logarithmic Transformation in the Presence of Some Zero Measurements eTable 3. Baseline Characteristics According to Whether Participants Had a 6-Week NNAL Measurement eTable 4. Effect of e-Cigarettes on Biomarkers of Exposure and Short-Term Cardiopulmonary Outcomes Adjusted for Marital Status eTable 5. Sensitivity Analysis Removing the People With a History of Chronic Obstructive Pulmonary Disease for Effect of e-Cigarettes on Biomarkers of Exposure and Short-Term Cardiopulmonary Outcomes eTable 6. Medians of Outcomes at Baseline, Week 2 and Week 6 by Study Randomization Group eTable 7. Medians of Outcomes at Baseline, Week 2 and Week 6 by Week Six Tobacco Use Trajectory eFigure 1. Patterns of Change in NNAL, co, and Cotinine for e-Cigarette vs Control Group eFigure 2. Patterns of Change in NNAL, co, and Cotinine for e-Cigarette Trajectory Groups [file jamanetwopen-e2026324-s002.pdf]

Supplemental Online Content

Pulvers K, Nollen NL, Rice M, et al. Effect of pod e-cigarettes vs cigarettes on carcinogen exposure among African American and Latinx smokers: a randomized clinical trial. *JAMA Netw Open*. 2020;3(11):e2026324. doi:10.1001/jamanetworkopen.2020.26324

eTable 1. Pod Flavor Selection of e-Cigarette Group Participants at Baseline and Week 2

eTable 2. Sensitivity Analysis for the Addition of a Constant to NNAL Measurements at Week 6 to Enable a Logarithmic Transformation in the Presence of Some Zero Measurements

eTable 3. Baseline Characteristics According to Whether Participants Had a 6-Week NNAL Measurement

eTable 4. Effect of e-Cigarettes on Biomarkers of Exposure and Short-Term Cardiopulmonary Outcomes Adjusted for Marital Status

eTable 5. Sensitivity Analysis Removing the People With a History of Chronic Obstructive Pulmonary Disease for Effect of e-Cigarettes on Biomarkers of Exposure and Short-Term Cardiopulmonary Outcomes

eTable 6. Medians of Outcomes at Baseline, Week 2 and Week 6 by Study Randomization Group

eTable 7. Medians of Outcomes at Baseline, Week 2 and Week 6 by Week Six Tobacco Use Trajectory

eFigure 1. Patterns of Change in NNAL, CO, and Cotinine for e-Cigarette vs Control Group

eFigure 2. Patterns of Change in NNAL, CO, and Cotinine for e-Cigarette Trajectory Groups

This supplemental material has been provided by the authors to give readers additional information about their work.

© 2020 Pulvers K et al. *JAMA Network Open.*

eTable 1. Pod Flavor Selection of e-Cigarette Group Participants at Baseline and Week 2

	All				Kansas City				San Diego			
**Timepoint**	Menthol	Mango	Cool Mint	Virginia Tobacco	Menthol	Mango	Cool Mint	Virginia Tobacco	Menthol	Mango	Cool Mint	Virginia Tobacco
Baseline N=125	44 (35.2)	35 (28.0)	24 (19.2)	22 (17.6)	38 (61.3)	0 (0.0)	12 (19.4)	12 (19.4)	6 (9.5)	35 (55.6)	12 (19.0)	10 (15.9)
Week 2[a] N=113	39 (34.5)	36 (31.9)	17 (15.0)	21 (18.6)	35 (60.3)	2 (3.4)	6 (10.3)	15 (25.9)	4 (7.3)	34 (61.8)	11 (20.0)	6 (10.9)

Notes. N (%) [a]Missing data for 12 participants (4 from Kansas City and 8 from San Diego) at Week 2.

© 2020 Pulvers K et al. *JAMA Network Open.*

eTable 2. Sensitivity Analysis for the Addition of a Constant to NNAL Measurements at Week 6 to Enable a Logarithmic Transformation in the Presence of Some Zero Measurements

**Constant**	**Within Group Change from Baseline**		**Between Group Change**
	**E-cig (*n* = 125)** Relative Risk (95% CI)	**Control (*n* = 61)** Relative Risk (95% CI)	**E-Cig vs. Control** Relative Risk (95% CI)
1.0	0.33 (0.26, 0.42) ***	0.92 (0.65, 1.30)	0.36 (0.23, 0.54) ***
0.2	0.28 (0.21, 0.38) ***	0.92 (0.61, 1.38)	0.31 (0.19, 0.51) ***
0.5	0.31 (0.24, 0.40) ***	0.92 (0.63, 1.34)	0.33 (0.21, 0.53) ***
0.8	0.32 (0.25, 0.41) ***	0.92 (0.64, 1.31)	0.35 (0.23, 0.54) ***
1.2	0.33 (0.26, 0.42) ***	0.92 (0.65, 1.30)	0.36 (0.24, 0.55) ***
1.5	0.34 (0.27, 0.43) ***	0.92 (0.66, 1.29)	0.37 (0.25, 0.56) ***

Notes: NNAL Normalized for creatinine; One sample missing; A constant of one was used for the primary analysis.
*** $p \le .001$; ** $p \le .01$; * $p \le .05$

© 2020 Pulvers K et al. *JAMA Network Open.*

eTable 3. Baseline Characteristics According to Whether Participants Had a 6-Week NNAL Measurement

Variable	Non-Missing<br>**(*n* = 168)**	Missing<br>**(*n* = 18)**
	Mean (SD) or N (%)	Mean (SD) or N (%)
Age	44.1 (12.5)	35.8 (9.4) **
Sex, % female	70 (41.7)	5 (27.8)
African American	90 (53.6)	2 (11.1) **
Latinx	78 (46.4)	16 (88.9) **
Education, ≤ high school	94 (56.0)	8 (44.4)
Income, ≤ 200% FPL	124 (73.8)	14 (77.8)
Marital status, never married	77 (45.8)	13 (72.2)
Menthol smoker, % yes	94 (56.0)	8 (44.4)
Number years smoking	17.0 (13.0)	15.7 (9.5)
Time to first cigarette ≤ 30 min	121 (72.0)	14 (77.8)
Days smoked/past 7[1]	6.8 (0.6)	6.9 (0.5)
Cigarettes per day/past 7[1]	12.3 (7.4)	10.5 (4.2)
Days used EC/past 7[1]	0.0 (0.3)	0.1 (0.5)
EC times on days used/past 7[1]	0.1 (0.5)	0.1 (0.4)
History of COPD, % yes	10 (6.0)	0
History of asthma, % yes	37 (22.0)	4 (22.2)
Mental health history, % any history[2]	97 (57.7)	10 (55.6)
History of substance abuse, % yes	80 (47.6)	8 (44.4)
Urine Cotinine, ng/mg[3]	1314.9 (1330.6)	1258.6 (1344.7)
Urine NNAL, pg/mg[3]	144.6 (121.5)	86.5 (79.5) *
Carbon monoxide (CO), ppm	18.5 (10.3)	16.4 (10.3)
Lung function, FEF 25-75%, L/sec[4]	3.0 (1.4)	3.6 (1.2)
Respiratory symptoms	11.2 (8.5)	11.7 (7.7)
Systolic blood pressure, mmHg	129.3 (17.0)	129.7 (14.6)
Diastolic blood pressure, mmHg	82.4 (10.1)	80.7 (10.9)

Notes: [1]From 7-day timeline follow-back; [2]Self-reported history of depression, anxiety, PTSD, or schizophrenia; [3]Normalized for creatinine; [4]Mean forced expiratory flow between 25% and 75% of forced vital capacity; *** p ≤ .001; ** p ≤ .01; * p ≤ .05

© 2020 Pulvers K et al. *JAMA Network Open.*

eTable 4. Effect of e-Cigarettes on Biomarkers of Exposure and Short-Term Cardiopulmonary Outcomes Adjusted for Marital Status

Variable	Within Group Change from Baseline		Between Group Change
	**E-cig (*n* = 125)** Relative Risk (95% CI)	**Control (*n* = 61)** Relative Risk (95% CI)	**E-Cig vs. Control** Relative Risk (95% CI)
**NNAL, pg/ml**[1]			
Week 6[2]	0.33 (0.26, 0.42) ***	0.92 (0.65, 1.30)	0.36 (0.23, 0.54) ***
**Cotinine, ng/ml**[1]			
Week 6	0.93 (0.78, 1.12)	1.17 (0.90, 1.53)	0.80 (0.58, 1.10)
**Carbon monoxide** (CO)**, ppm**			
Week 2	0.45 (0.38, 0.52) ***	0.98 (0.79, 1.22)	0.45 (0.35, 0.59) ***
Week 6	0.48 (0.41, 0.55) ***	0.90 (0.73, 1.09)	0.53 (0.42, 0.68) ***
**Cigarettes, past 7 days**[3]			
Week 2	0.23 (0.18, 0.30) ***	0.95 (0.73, 1.23)	0.25 (0.17, 0.36) ***
Week 6	0.23 (0.18, 0.30) ***	0.77 (0.59, 1.01)	0.30 (0.20, 0.43) ***
**Respiratory symptoms, per point**			
Week 2	0.90 (0.78, 1.05)	1.20 (0.97, 1.47)	0.76 (0.59, 0.97) *
Week 6	0.70 (0.60, 0.83) ***	1.11 (0.87, 1.42)	0.63 (0.47, 0.85) **
**Lung function, FEF 25-75%, L/sec**[4]			
Week 2	0.99 (0.92, 1.07)	1.01 (0.91, 1.12)	0.98 (0.87, 1.12)
Week 6	0.96 (0.88, 1.04)	1.01 (0.89, 1.14)	0.95 (0.82, 1.11)
**Systolic blood pressure, mmHg**			
Week 2	1.00 (0.98, 1.02)	1.01 (0.98, 1.04)	0.99 (0.96, 1.03)
Week 6	1.01 (0.98, 1.03)	1.03 (0.99, 1.06)	0.98 (0.94, 1.02)
**Diastolic blood pressure, mmHg**			
Week 2	0.99 (0.97, 1.02)	1.00 (0.97, 1.03)	1.00 (0.96, 1.04)
Week 6	1.00 (0.98, 1.02)	1.00 (0.97, 1.03)	1.00 (0.97, 1.04)

Notes: [1]Normalized for creatinine; [2]One sample missing; [3]From 7-day timeline follow-back, continuing smokers only (n= 126); [4]Mean forced expiratory flow between 25% and 75% of forced vital capacity; *** $p \le .001$; ** $p \le .01$; * $p \le .05$

© 2020 Pulvers K et al. *JAMA Network Open.*

eTable 5. Sensitivity Analysis Removing the People With a History of Chronic Obstructive Pulmonary Disease for Effect of e-Cigarettes on Biomarkers of Exposure and Short-Term Cardiopulmonary Outcomes

Variable	**Within Group Change from Baseline**		**Between Group Change**
	**E-cig (*n* = 125)** Relative Risk (95% CI)	**Control (*n* = 61)** Relative Risk (95% CI)	**E-Cig vs. Control** Relative Risk (95% CI)
**NNAL, pg/ml**[1]			
Week 6[2]	0.32 (0.25, 0.42) ***	0.92 (0.65, 1.30)	0.35 (0.23, 0.54) ***
**Cotinine, ng/ml**[1]			
Week 6	0.94 (0.77, 1.14)	1.17 (0.89, 1.53)	0.80 (0.58, 1.12)
**Carbon monoxide** (CO), **ppm**			
Week 2	0.45 (0.38, 0.52) ***	0.98 (0.79, 1.22)	0.46 (0.35, 0.60) ***
Week 6	0.48 (0.41, 0.55) ***	0.89 (0.73, 1.09)	0.54 (0.42, 0.69) ***
**Cigarettes, past 7 days**[3]			
Week 2	0.22 (0.16, 0.28) ***	0.95 (0.73, 1.23)	0.23 (0.16, 0.33) ***
Week 6	0.23 (0.17, 0.30) ***	0.77 (0.59, 1.01)	0.29 (0.20, 0.43) ***
**Respiratory symptoms, per point**			
Week 2	0.91 (0.78, 1.06)	1.20 (0.97, 1.47)	0.76 (0.59, 0.98) *
Week 6	0.73 (0.61, 0.87) ***	1.11 (0.88, 1.42)	0.65 (0.49, 0.88) **
**Lung function, FEF 25-75%, L/sec**[4]			
Week 2	1.00 (0.92, 1.07)	1.01 (0.91, 1.11)	0.99 (0.87, 1.12)
Week 6	0.96 (0.88, 1.05)	1.00 (0.89, 1.14)	0.96 (0.82, 1.11)
**Systolic blood pressure, mmHg**			
Week 2	1.00 (0.98, 1.02)	1.01 (0.98, 1.04)	0.99 (0.95, 1.03)
Week 6	1.01 (0.98, 1.03)	1.03 (0.99, 1.06)	0.98 (0.94, 1.02)
**Diastolic blood pressure, mmHg**			
Week 2	0.99 (0.97, 1.02)	1.00 (0.97, 1.03)	0.99 (0.96, 1.04)
Week 6	1.00 (0.98, 1.02)	1.00 (0.97, 1.03)	1.00 (0.96, 1.04)

Notes: [1]Normalized for creatinine; [2]One sample missing; [3]From 7-day timeline follow-back, continuing smokers only (n= 121); [4]Mean forced expiratory flow between 25% and 75% of forced vital capacity; *** $p \leq .001$; ** $p \leq .01$; * $p \leq .05$

© 2020 Pulvers K et al. *JAMA Network Open.*

eTable 6. Medians of Outcomes at Baseline, Week 2 and Week 6 by Study Randomization Group.

Variable	EC Group			Cigarettes as Usual Control Group		
	**Baseline (*n* = 125)**	**Week 2 (*n* = 114)**	**Week 6 (*n* = 114)**	**Baseline (*n* = 61)**	**Week 2 (*n* = 57)**	**Week 6 (*n* = 54)**
Urine NNAL, pg/ml[1]	124 (45, 197)[a]	--	40 (12, 101)	88 (58, 197)	--	97 (39, 222)
Urine Cotinine, ng/ml[1]	928 (463, 1476)[b]	--	835 (476, 1334)	1061 (534, 1720)	--	1289 (643, 2078)
Carbon monoxide (CO), ppm	16 (11, 22)	7 (3, 14)	7 (3, 14)	17 (11, 25)	16 (11, 28)	16 (9, 25)
Cigarettes, past 7 days[2] Full sample	72 (49, 110)	3 (0, 27)	2 (0, 18)	70 (45, 105)	63 (48, 91)	58 (35, 77)
Cigarettes, past 7 days[2] Continuing smokers	84 (49, 116)[c]	27 (7, 49)[d]	18 (7, 49)[g]	70 (45, 105)	63 (48, 91)	58 (35, 77)
Respiratory symptoms, per point	11 (5, 18)[b]	9 (5, 15)[e]	7 (3, 12)[a]	8 (4, 13)	10 (4, 16)[b]	9 (4, 16)[b]
Lung function, FEF 25-75%, L/sec[3]	3.0 (2.1, 4.1)[b]	3.0 (2.0, 4.1)[f]	2.8 (1.8, 4.1)	2.8 (2.1, 4.0)	2.7 (2.2, 3.9)	2.7 (2.1, 4.1)[b]
Systolic blood pressure, mmHg	130 (115, 142)	129 (115, 144)	128 (117, 142)	129 (118, 140)	130 (117, 144)	133 (122, 143)
Diastolic blood pressure, mmHg	81 (76, 89)	81 (75, 89)	83 (74, 89)	83 (74, 88)	83 (74, 88)	83 (75, 89)

Notes: Median (25%, 75%). [1]Normalized for creatinine; [2]From 7-day timeline follow-back; [3]Mean forced expiratory flow between 25% and 75% of forced vital capacity; [a]Two samples missing; [b]One sample missing; [c]Sub-sample *n* = 65; [d]Sub-sample *n* = 54; [e]Four samples missing; [f]Three samples missing; [g]Sub-sample *n* =56

© 2020 Pulvers K et al. *JAMA Network Open.*

eTable 7. Medians of Outcomes at Baseline, Week 2 and Week 6 by Week Six Tobacco Use Trajectory

Variable	EC Only				Dual EC+Cig				Cig Only			
	**Baseline[1] (*n* = 32)**	**Week 2 (*n* = 32)**	**Baseline[2] (*n* = 32)**	**Week 6 (*n* = 32)**	**Baseline[1] (*n* = 77)**	**Week 2 (*n* = 77)**	**Baseline[2] (*n* = 66)**	**Week 6 (*n* = 66)**	**Baseline[1] (*n* = 4)**	**Week 2 (*n* = 4)**	**Baseline[2] (*n* = 16)**	**Week 6 (*n* = 16)**
Urine NNAL, pg/ml[3]	--	--	141 (66, 200)	7 (3, 23)	--	--	108 (44, 203)	47 (22, 103)	--	--	132 (57, 187)[a]	100 (70, 273)
Urine Cotinine, ng/ml[3]	--	--	907 (377, 1551)	928 (525, 1409)	--	--	897 (474, 1363)	699 (441, 1090)	--	--	1081 (469, 1686)[a]	1034 (836, 1502)
Carbon monoxide (CO), ppm	16 (10, 20)	3 (2, 3)	18 (14, 23)	3 (2, 3)	17 (12, 23)	12 (5, 16)	15 (11, 22)	10 (6, 14)	13 (12, 17)	9 (8, 11)	15 (12, 20)	17 (13, 30)
Cigarettes, past 7 days[4]	67 (49, 92)	0 (0, 0)	70 (46, 108)	0 (0, 0)	70 (48, 116)	11 (2, 34)	73 (48, 105)	5 (1, 18)	79 (60, 92)	88 (67, 110)	87 (50, 129)	70 (20, 90)
Respiratory symptoms, per point	6 (4, 20)	6 (4, 10)	9 (5, 17)	6 (2, 9)[a]	12 (6, 17)[a]	10 (5, 15)[b]	11 (6, 19)	9 (4, 14)	14 (7, 20)	19 (15, 20)	8 (5, 14)[a]	10 (7, 14)[a]
Lung function, FEF 25-75%, L/sec[5]	3.3 (2.6, 4.3)	2.9 (1.9, 4.2)	3.0 (2.1, 4.1)	2.7 (1.9, 4.0)	2.8 (2.0, 3.8)[a]	3.1 (2.0, 4.1)[c]	3.0 (2.0, 4.3)	2.7 (1.7, 4.3)	4.4 (2.9, 5.6)	2.7 (1.4, 4.2)	2.8 (2.1, 3.7)	2.9 (1.9, 3.4)
Systolic blood pressure, mmHg	121 (114, 134)	120 (113, 133)	124 (114, 133)	123 (114, 133)	133 (120, 144)	132 (119, 146)	132 (115, 143)	129 (118, 142)	125 (105, 146)	124 (110, 141)	136 (125, 158)	130 (122, 155)

© 2020 Pulvers K et al. *JAMA Network Open.*

Diastolic blood pressure, mmHg	78 (72, 90)	79 (75, 87)	80 (75, 90)	80 (75, 86)	82 (78, 89)	83 (75, 89)	82 (76, 88)	83 (73, 89)	83 (69, 97)	82 (75, 89)	87 (79, 95)	85 (79, 97)

Notes: Median (25%, 75%). [1]Baseline comparison group matched to those in same tobacco use category at week 2. [1]Baseline comparison group matched to those in same tobacco use category at week 6. [3]Normalized for creatinine; [4]From 7-day timeline follow-back, full sample; [5]Mean forced expiratory flow between 25% and 75% of forced vital capacity; [a]One sample missing; [b]Four samples missing; [c]Three samples missing

© 2020 Pulvers K et al. *JAMA Network Open.*

eFigure 1. Patterns of Change in NNAL, CO, and Cotinine for e-Cigarette vs Control Group

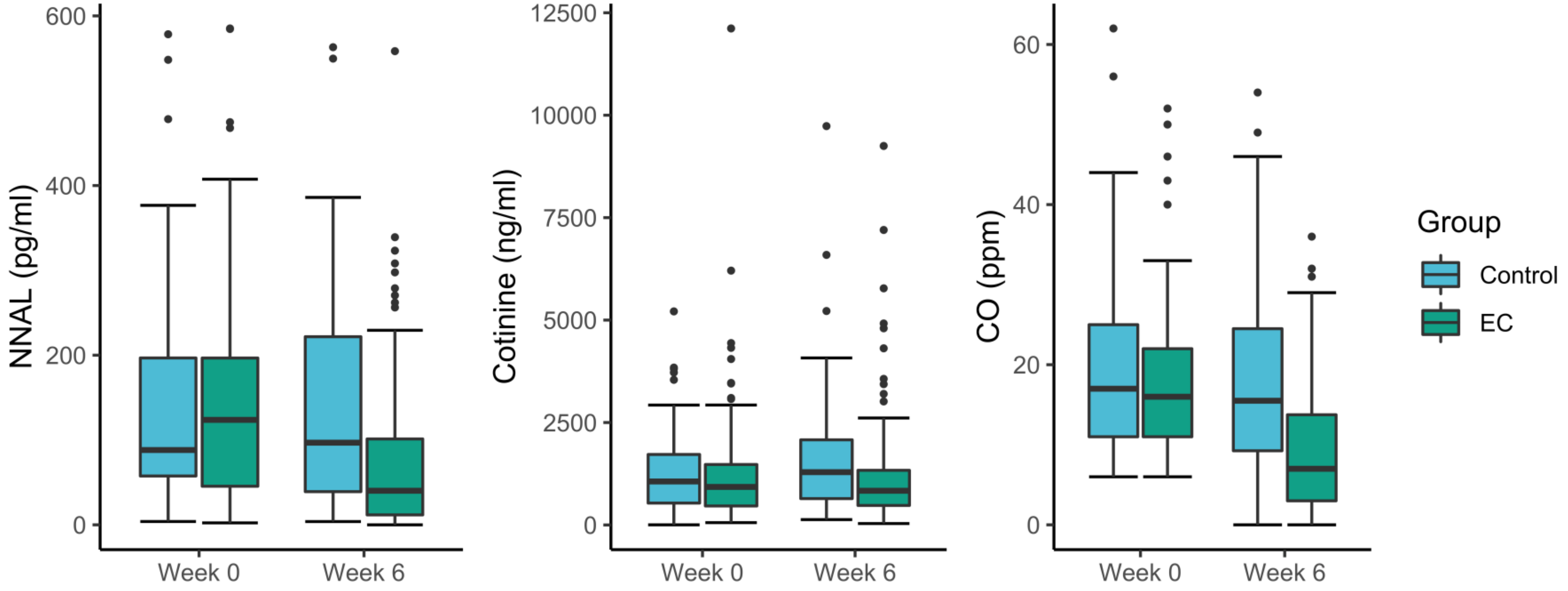

Notes: Median and interquartile range (25%-75%) reported. NNAL pg/ml and Cotinine ng/ml normalized for creatinine. Carbon monoxide (CO) reported in PPM. Significant between group changes for NNAL and CO, $p < .001$.

© 2020 Pulvers K et al. *JAMA Network Open.*

eFigure 2. Patterns of Change in NNAL, CO, and Cotinine for e-Cigarette Trajectory Groups

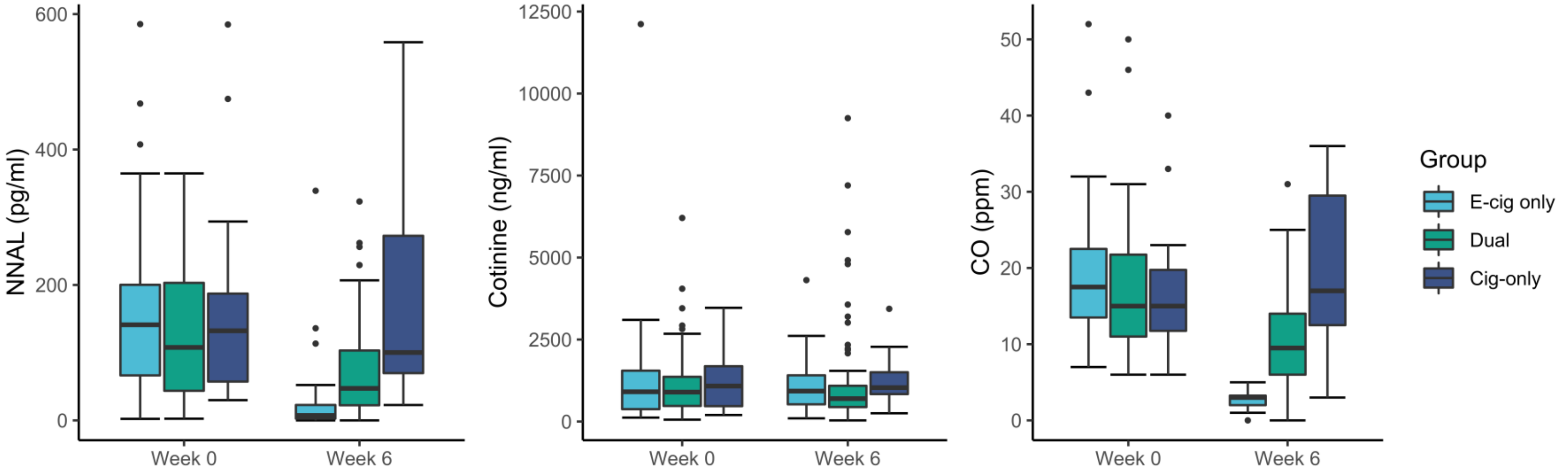

Notes: Median and interquartile range (25%-75%) reported. NNAL pg/ml and Cotinine ng/ml normalized for creatinine. Carbon monoxide (CO) reported in ppm.

Classification for EC trajectory groups at week 6: E-cig (EC) only is any use of ECs, no use of cigarettes in the past 7 days, and CO < 6 ppm; Those who reported no use of cigarettes in the past 7 days but who had a CO ≥ 6 were classified as dual users. Dual is any use of ECs and any use of cigarettes in the past 7 days; and Cig-only is no use of ECs and any use of cigarettes in the past 7 days.

Significant between group changes. NNAL: E-cig only vs Cig-only and E-cig only vs Dual, $p < .001$. Cotinine: None. CO: E-cig only vs Cig-only and E-cig only vs Dual, $p < .001$; Dual vs Cig-only $p < .01$.

© 2020 Pulvers K et al. *JAMA Network Open.*
