# Supplementary material for: Effect of Pod e-Cigarettes vs Cigarettes on Carcinogen Exposure Among African American and Latinx Smokers: A Randomized Clinical Trial
Source: JAMA Netw Open. 2020 Nov 18;3(11):e2026324. doi: 10.1001/jamanetworkopen.2020.26324 (PMC7675102; doi:10.1001/jamanetworkopen.2020.26324)
Supplement: Supplement 3. — Data Sharing Statement [file jamanetwopen-e2026324-s003.pdf]

Data Sharing Statement

Pulvers. Effect of Pod e-Cigarettes vs Cigarettes on Carcinogen Exposure Among African American and Latinx Smokers. *JAMA Netw Open*. Published November 18, 2020.
10.1001/jamanetworkopen.2020.26324

Data
Data available: No
